# Thrombocytopenia and Hemostatic Changes in Acute and Chronic Liver Disease: Pathophysiology, Clinical and Laboratory Features, and Management

**DOI:** 10.3390/jcm10071530

**Published:** 2021-04-06

**Authors:** Rüdiger E. Scharf

**Affiliations:** 1 Program in Cellular and Molecular Medicine, Boston Children’s Hospital, Harvard Medical School, Boston, MA 02115, USA; 2 Division of Experimental and Clinical Hemostasis, Hemotherapy, and Transfusion Medicine, Blood and Hemophilia Comprehensive Care Center, Institute of Transplantation Diagnostics and Cell Therapy, Heinrich Heine University Medical Center, D-40225 Düsseldorf, Germany; rscharf@uni-duesseldorf.de

**Keywords:** advanced liver disease, bleeding risk, cirrhosis, hemostasis, thrombocytopenia, thrombopoietin receptor agonists/mimetics

## Abstract

Thrombocytopenia, defined as a platelet count <150,000/μL, is the most common complication of advanced liver disease or cirrhosis with an incidence of up to 75%. A decrease in platelet count can be the first presenting sign and tends to be proportionally related to the severity of hepatic failure. The pathophysiology of thrombocytopenia in liver disease is multifactorial, including (*i*) splenomegaly and subsequently increased splenic sequestration of circulating platelets, (*ii*) reduced hepatic synthesis of thrombopoietin with missing stimulation both of megakaryocytopoiesis and thrombocytopoiesis, resulting in diminished platelet production and release from the bone marrow, and (*iii*) increased platelet destruction or consumption. Among these pathologies, the decrease in thrombopoietin synthesis has been identified as a central mechanism. Two newly licensed oral thrombopoietin mimetics/receptor agonists, avatrombopag and lusutrombopag, are now available for targeted treatment of thrombocytopenia in patients with advanced liver disease, who are undergoing invasive procedures. This review summarizes recent advances in the understanding of defective but at low level rebalanced hemostasis in stable cirrhosis, discusses clinical consequences and persistent controversial issues related to the inherent bleeding risk, and is focused on a risk-adapted management of thrombocytopenia in patients with chronic liver disease, including a restrictive transfusion regimen.

## 1. Introduction

Complex disorders of the hemostatic apparatus are present in acute and chronic liver disease, involving combined abnormalities of the megakaryocyte-platelet system, coagulation, and fibrinolysis. Apart from coagulation defects (international normalized ratio, INR > 1.5) due to reduced hepatocellular synthetic capacity, thrombocytopenia of variable extent is a frequent feature both in acute and chronic liver disease. Importantly, a decrease in platelet count tends to occur prior to clinical manifestations associated with progressive liver failure and decompensation. Thus, thrombocytopenia can be a sensitive noninvasive biomarker of liver disease and be used as a clinical diagnostic tool.

This review covers several aspects of platelet pathology in the context of a defective but at low level rebalanced hemostasis in stable cirrhosis, addresses clinical features and controversial issues, and discusses progress in the management of patients with chronic liver disease and associated risks.

## 2. Incidence of Thrombocytopenia

An analysis by the Acute Liver Failure Study Group, enrolling 1600 patients, documented that the median platelet count on admission was approximately 130,000/μL; 60% of patients had platelets counts <150,000/μL, 35% <100,000/μL, and 10% <50,000/μL [1]. Despite a perceived hemorrhagic diathesis, clinically significant bleeding is rather uncommon in acute liver disease. This observation is confirmed by a recent analysis on adult 1770 patients with acute liver failure by a Dutch Study Group [2]. Despite a median INR of 2.7 and platelet count of 96,000/μL on admission, hemorrhagic complications occurred in only 187 patients (11%), including 173 spontaneous and 22 postprocedural bleeding events. However, 20 subjects among this patient cohort experienced an intracranial hemorrhage [2]. Progressive thrombocytopenia in acute liver disease can be an indicator of impending multi-organ failure, as documented in a retrospective study [3]. Specifically, an early decrease in platelets (during days 1 to 7 after admission) was proportional to the grade of hepatic encephalopathy, requirement for treatment with vasopressor agents, and kidney replacement therapy [3].

In patients with advanced fibrosis or liver cirrhosis, the prevalence of thrombocytopenia ranges between 15 and 75% [4,5,6]. A progressive decrease in platelet count is considered as a noninvasive indicator for the development of portal hypertension due to severe liver fibrosis or cirrhosis [7]. Overall, the degree of thrombocytopenia appears to be proportionally related to the severity of liver disease but is not associated with spontaneous bleeding, unless platelets counts decrease to <50,000–60,000/μL [8,9,10,11,12,13].

## 3. Is Thrombocytopenia a Predictive Parameter of the Bleeding Risk in Chronic Liver Disease?

Several investigators have suggested that thrombocytopenia may be of predictive validity regarding hemorrhagic events in patients with cirrhosis. However, data on the overall significance of lowered platelet counts among this patient population are equivocal. Thus, a number of studies indicate severe thrombocytopenia (<50,000/μL) to be a predictor of major bleeding and re-bleeding in the peri-interventional setting [14], while others cannot confirm an explicit correlation between low platelet counts and the incidence of periprocedural hemorrhage [6,15]. The conflicting conclusions result, at least in part, from differences in study populations and procedures.

## 4. Pathophysiology

Thrombocytopenia in liver disease results from increased splenic sequestration due to portal hypertension and subsequent hypersplenism [7,8,13,16], decreased thrombopoietin (TPO) production [6,17,18,19], and/or from toxic or virus-induced suppression of megakaryocytopoiesis [6,19,20,21]. In the past, low platelet counts in liver disease were mainly attributed to splenic pooling and/or consumption. By contrast, autoantibody-mediated platelet destruction is of minor importance in most cases with chronic liver disease, but may be relevant in hepatitis C-induced cirrhosis [6]. In these patients, antiplatelet antibodies are detectable, often at high levels [22]. More recently, the impact of abnormal rheological conditions, resulting from enhanced portal pressure, is also discussed as an additional mechanism of increased platelet destruction in chronic liver disease [19]. It is hypothesized that platelets, upon exposure to high shear stress and subsequent activation, are rapidly eliminated from the circulation, thereby potentiating thrombocytopenia. However, this contention is distinct from established high-shear conditions resulting in bleeding complications due to loss of high-molecular-weight von Willebrand factor [23].

During the past decades, detailed exploration of the association between hepatic synthesis of TPO and residual hepatic function allowed a more specific insight into the pathophysiology of thrombocytopenia in liver disease. In fact, TPO levels are near-normal or even increased in acute hepatic disease [18]. This is in contrast to patients with chronic liver disease, in whom TPO serum levels are significantly decreased and therefore thought to be a central pathomechanism to thrombocytopenia in cirrhosis [6,24]. TPO is predominantly synthesized in hepatocytes and, consequently, reduced upon damage to or destruction of liver cell mass. Importantly, among other cytokines involved, TPO is the only one, driving both megakaryocytopoiesis and thrombocytopoiesis at all stages of differentiation and maturation (i.e., from stem cell to multipotent progenitor committed megakaryocyte progenitor cell, immature and mature megakaryocyte to the formation and release of platelets) [25]. The availability of TPO receptor agonists in patients with chronic liver disease and severe thrombocytopenia has therefore been highly anticipated [26,27]. Details on the use of the agents are discussed in the Management Section 9.

## 5. “Low-Level” Hemostasis—A Defective but Rebalanced System in Liver Disease

**Figure 1:**
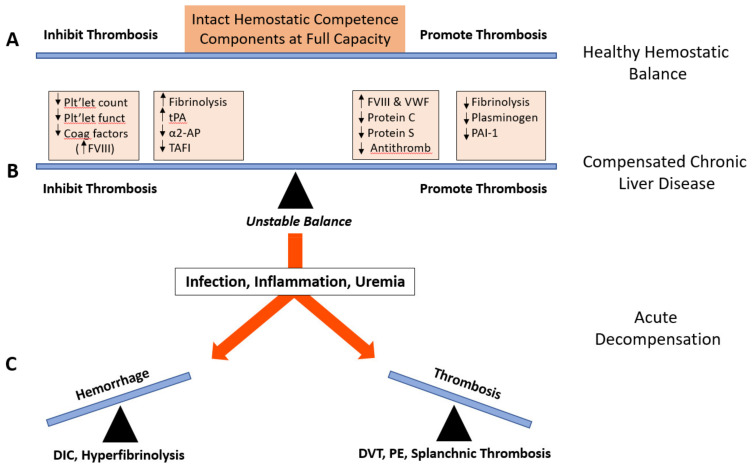
The concept of rebalanced hemostasis in patients with liver disease. In healthy subjects (**A**), hemostasis is in stable equilibrium. In patients with liver disease (**B**), concomitant changes in pro- and antihemostatic components and pathways result in rebalance of the hemostatic apparatus despite the quantitative and qualitative disease-related defects, affecting platelets (primary hemostasis), coagulation (secondary hemostasis), and fibrinolysis. Thus, hemostasis is rebalanced at low level, but the equilibrium now is extremely labile. In patients with decompensated liver disease (**C**) and comorbidities, various stimuli can lead to destabilization and tip the balance toward either bleeding or thrombosis [29]. Modification of a scheme, taken from Eby and Caldwell [31]. Abbreviations: α_2_-AP, α_2_-antiplasmin; Antithromb, antithrombin; DIC, disseminated intravascular coagulation; DVT, deep vein thrombosis; F, factor; PAI-1, plasminogen activator inhibitor-1; PE, pulmonary embolism; Plt’let, platelet; TAFI, thrombin activatable fibrinolysis inhibitor; t-PA, tissue-type plasminogen activator; VWF, von Willebrand factor.

Despite the multifaceted hemostatic defects, bleeding episodes due to compromised hemostasis are relatively infrequent in patients with liver disease. Based on clinical and systematic laboratory findings, we hypothesized as early as the mid-1980s that a balanced *low-level* hemostatic equilibrium due to a *concordant* reduction in pro- and anti-hemostatic components is present in stable liver cirrhosis [8,16]. However, this balance is extremely labile and thus can be easily destabilized by various triggers (e.g., infection, variceal bleeding, decompensated liver cirrhosis, invasive procedures, or inadequate hemotherapy with prothrombotic components such fresh-frozen plasma, activated prothrombin complex concentrates, or recombinant factor VIIa). More recent studies have confirmed our contention of a *rebalanced* hemostasis (Figure 1), resulting from a commensurate decline in pro- and anti-hemostatic factors both in patients with acute and chronic liver disease [28,29,30].

Moreover, despite decreased procoagulant coagulation factors, thrombocytopenia and suspected platelet dysfunction, patients with acute or chronic liver disease can display hypercoagulable features, which may explain, at least in part, that thrombotic complications are more common than spontaneous bleeding complications [28,29,30]. For example, apart from normal or even enhanced thrombin generation in liver cirrhosis [12,32,33], Stravitz et al., have shown increased levels of highly procoagulant platelet-derived microparticles [34].

In addition, elevated plasma levels of von Willebrand factor (VWF), typically observed in chronic liver disease, can compensate for the low numbers of circulating platelets [35] and may restore primary hemostasis [28,30,32]. Concomitantly, concentrations of ADAMTS13, a plasma metalloprotease that cleaves high-molecular-weight VWF species into smaller and less prohemostatic VWF multimers, are decreased in patients with cirrhosis and thus support VWF-mediated platelet adhesion to the subendothelium at sites of vascular lesions [32,36]. However, these compensatory mechanisms fail to operate in patients with end-stage liver disease (Figure 1), in whom prominent bleeding complications remain a serious concern [32].

## 6. Platelet Dysfunction in Liver Cirrhosis

Concomitant platelet function defects, as suggested in chronic liver disease, are less well defined [23]. Generally, it has been assumed that platelet function deteriorates with the severity of liver disease. Older studies have described in vitro aggregation abnormalities in response to several agonists [37,38]. Some of the platelet aggregation defects were attributed to elevated levels of fibrinogen/fibrin degradation products or dysfibrinogenemia, both of which are rather common in chronic hepatitis and cirrhosis. Combined α- and δ-granule storage pool deficiency, as reported in a small series of patients [39], can impair platelet function and favor a hemorrhagic diathesis in chronic liver disease. However, platelets from patients with severe but stable cirrhosis display a normal content of α- and δ-granule constituents [8,16]. Thus, if at all, storage pool deficiency does not appear to play a significant role in the pathogenesis of platelet defects in cirrhotic patients. Importantly, toxic effects of ethanol may also contribute to platelet dysfunction [20]. Other possible mechanisms include defects in the glycoprotein (GP) Ib of the GPIb-IX-V complex [40], decreased availability of arachidonic acid and consequently reduced biosynthesis of thromboxane A_2_ [41], increased cholesterol content of the platelet plasma membrane, impaired transmembrane signaling [41], elevated sialic acid concentration of circulating platelets, and hypersialylated fibrinogen [8].

Platelet defects resulting from various mechanisms, as outlined above, may remain compensated and clinically inapparent as long as platelet function (and/or coagulation) is not inhibited pharmacologically [42]. Therefore, drug-induced platelet dysfunction is a major concern, specifically in thrombocytopenic patients with liver cirrhosis.

By contrast, antiplatelet antibodies, rather infrequently found in patients with acute or chronic viral liver disease, appear to be of minor relevance in this setting with regard to qualitative or quantitative platelet disorders [4]. The diagnosis of impending disseminated intravascular coagulation (DIC) in patients with liver disease is often difficult to ascertain because of the multiple hemostatic alterations.

## 7. Clinical Features of Bleeding in Liver Disease

Bleeding is common among patients with liver disease but less frequent than generally assumed and also emphasized in traditional textbooks. Hemorrhagic diathesis includes spontaneous hematomas, oozing from oropharyngeal mucosa, and bleeding upon dental extraction, skin puncture, or from biopsy sites. Compared with age-matched individuals, the incidence of non-variceal upper gastrointestinal hemorrhage in patients with liver disease is estimated to be twice as high as in the general population (50–100 per 100,000 person per year) [43]. By contrast, intracranial bleeding is rare.

Given the fact that about 50% of cirrhotic patients develop gastroesophageal varices (and that one-third will experience variceal hemorrhage), patients with chronic liver disease are at risk for bleeding complications. This is illustrated by a high in-hospital mortality rate of approximately 15% for each episode of acute gastrointestinal hemorrhage, and variceal bleeding is the cause of death for approximately 6% of patients with liver cirrhosis [31]. Predictors of a first upper gastrointestinal bleeding in cirrhotic patients include presence of varices, elevated variceal pressure, and laboratory evidence of decreased hepatocellular synthesis (with or without manifest coagulopathy), and thrombocytopenia.

Moderate or severe bleeding complications reported after percutaneous liver biopsy are consistently low with rates of 0.5 to 0.7% in large contemporary series [31,44]. However, these findings should be considered with caution since most of the data from these studies are derived from patients in relatively stable or compensated condition of advanced liver disease. This is also true for attempts to correlate hemostasis test results with clinical outcome or prediction of bleeding in the setting of invasive procedures.

Drugs represent the most common cause of acquired platelet dysfunction in our overmedicated society. Apart from typical antiplatelet agents such as aspirin, adenosine diphosphate receptor antagonists, and integrin αIIbβ3 (GPIIb-IIIa) receptor blockers, other widely used agents, including non-steroidal anti-inflammatory drugs, antibiotics, cardiovascular and lipid-lowering drugs, selective serotonin reuptake inhibitors, and a plethora of miscellaneous agents, diets, and food additives or spices can affect platelet function and cause or aggravate a hemorrhagic diathesis [42].

## 8. Laboratory Assessment of Bleeding Risk in Liver Disease

### 8.1. Hemostasis Screening Tests—Often Inconclusive

Analysis of hemostasis in patients with advanced liver disease remains crucial for correct diagnosis, management decisions, assessment of bleeding risk, and monitoring of treatment, specifically of hemotherapy. However, most routine laboratory hemostasis tests are not really suitable to predict bleeding. For example, prolongation of bleeding time is found in about 40% of cirrhotic patients but a poor predictor of hemorrhage after percutaneous liver biopsy [31]. Moreover, screening coagulation tests are imprecise and display potentially misleading results in advanced liver disease. In particular, prothrombin time (INR) is of limited predictive value with regard neither to the bleeding nor to the thrombotic risk in a given patient. Thus, an elevated INR only reflects a reduction in procoagulant coagulation factors due to decreased hepatocellular synthesis but not the concomitant decline in anticoagulant activity of the protein C pathway. Alternative methods such as thrombin generation assays might not be routinely available and have not been sufficiently validated to be recommended as replacements for INR. This is in contrast to thromboelastography (TEG), a viscoelastic assay that more closely reflects global hemostasis in vivo. Importantly, TEG may overcome some of the limitations or shortcomings of standard coagulation tests in patients with acute and chronic liver disease [45].

#### 8.1.1. Thromboelastography

TEG allows to dissect the kinetic conversion of fibrinogen into fibrin, the dynamics of fibrin- and platelet-driven clot formation, and the assessment of clot strength and clot stability. TEG can also be helpful for detecting abnormal fibrinolysis.

TEG is now widely used in patients with acute and chronic liver disease, specifically in subjects undergoing liver transplantation to guide replacement therapy with hemostatic factor concentrates (during the pre-anhepatic and anhepatic phases) and to monitor treatment of hyperfibrinolysis. However, neither the TEG method nor the hemotherapeutic consequences for correction of TEG parameters are consistently standardized across transplantation centers [45].

Interestingly, patients with cirrhosis secondary to cholestatic liver disease such as primary biliary cirrhosis (PBC) or primary sclerosing cholangitis (PSC) have been found to be hypercoagulable by TEG when compared to patients with noncholestatic entities [46,47]. This difference may be due to higher levels of fibrinogen and a still intact platelet function in the cholestatic stetting despite similar degrees of portal hypertension in the different patient populations [48]. Taken together, these observations may explain the lower rates of bleeding complications and fewer transfusion needs during liver transplantation in patients with PBC or PSC than in those with end-stage liver disease of other etiology. Moreover, several studies have demonstrated that TEG parameters indicative of hypocoagulation and/or thrombocytopenia are associated with liver disease severity and outcomes [49,50,51].

#### 8.1.2. Standardization and Validation of Whole Blood Viscoelastic Assays

Most investigations suggest that analysis of patients with acute and chronic liver disease by TEG provides a more reliable assessment of the hemorrhagic risk than standard coagulation assays and determination of the bleeding time. However, only a few studies have directly compared the results of standard coagulation and viscoelastic assays. Overall, a poor correlation between test parameters of both approaches was reported [52].

During the past years, attempts have been made to implement standardization of viscoelastic testing in a variety of clinical conditions including acute and chronic liver disease [53]. Two commercially devices are currently used: the TEG^TM^ (TEG500 and TEG6s) and ROTEM (with a number of different modules including ROTEM platelet). The clinical utility and reliability of thromboelastography have recently been assessed in a prospective validation study [54]. Moreover, thromboelastographic reference ranges within a population of cirrhotic patients undergoing liver transplantation are now proposed [55].

### 8.2. MELD Score

The model for end-stage liver disease (MELD), initially created in 2000 by investigators from the Mayo Clinic to predict survival in patients with portal hypertension undergoing placement of transjugular intrahepatic portosystemic shunts (TIPS) [56], and subsequent refinement modifications of the MELD score are being broadly used to predict survival and bleeding complications in a variety of patient cohorts with liver disease of different etiology and distinct interventions [57,58]. MELD incorporates three widely available variables (INR, serum creatinine, and serum bilirubin). Thus, the score is affected by variability and interlaboratory variation of INR determinations [31], which in turn specifically limits its application to predict bleeding complications along with invasive procedures [59]. Consequently, most physicians are left in the difficult position of relying on clinical assessment of the individual bleeding risk when deciding whether or not to administer potentially harmful hemotherapy or hemostatic treatment.

### 8.3. Platelet Thresholds—Not Based on High-Quality Data

By contrast to screening coagulation tests, thrombocytopenia can be an indicator of increased hemorrhagic risk in patients with liver disease. As documented by a number of retrospective case series [31,60,61], bleeding complication rates after percutaneous liver biopsies are higher at platelet counts <50,000/μL. Based on these data, most recognized centers in Europe require preprocedural platelet counts >80,000/μL, whereas a survey of US centers showed a preference for a platelet threshold of >50,000/μL [60]. Thus, the evidence for a valid “cutoff” value remains scanty.

A very recent re-evaluation of published studies has revealed the paucity of data to recommend a “safe” minimum platelet number for invasive procedures in patients with chronic liver disease [61]. Importantly, in a large retrospective study of patients having percutaneous liver biopsy, implementation of less stringent preprocedural hemostasis parameters (INR < 2.0, platelet counts >25,000/μL) was associated with fewer hemorrhagic complication rates and a decrease in preprocedural administration of fresh-frozen plasma and platelet concentrates in comparison with using “historical” cut-off levels (INR < 1.5, platelet counts > 50,000/μL) [62]. A possible explanation for this apparent contradiction is that INR and platelet counts are surrogate markers of liver fibrosis and/or portal hypertension, which per se may be risk factors for bleeding. Such a contention would provide a plausible reason that attempts to correct hemostatic changes can increase the hemorrhagic risk since defective hemostasis is not necessarily the underlying cause of bleeding, and increasing the intravascular volume is likely to be counterproductive [61]. As discussed below, transfusion-associated circulatory overload is a major concern in the management of patients with chronic liver disease.

## 9. Management of Thrombocytopenia in Patients with Liver Disease

Generally, the approach to bleeding in patients with acute or chronic hepatic disease can be divided into supportive measures, prophylactic treatment prior to invasive procedures, and in rescue therapy for active bleeding. In particular, management of thrombocytopenia in cirrhotic patients is a challenging task for physicians both in the in- and out-patient setting.

### 9.1. Hemotherapy with Platelet Concentrates

Until recently, transfusion of platelets has been a gold standard for the management of thrombocytopenia in liver disease patients. Generally, two types of platelet concentrates are available for hemotherapy: single-donor apheresis platelet concentrates (SDAPC) and pooled whole blood-derived platelet concentrates (PPC) that are obtained from 4 to 6 donors and prepared either using the platelet rich plasma (PRP-PC) or the buffy coat (BC-PC) method [63,64,65]. There is ongoing debate on whether or not SDAPC are superior to PPC, specifically with regard to individuals, who frequently require multiple platelet transfusions such as cirrhotic patients [66,67,68,69,70]. Of note, SDAPC are preferentially used in France, Germany, and the UK, whereas in the USA more than 90% of platelet transfusions are performed using pooled PRP-PC.

### 9.2. Limitations of Platelet Transfusions

Despite advances in pathogen safety, platelet collection, preparation technologies, and storage modalities, potential risks associated with hemotherapy persist, including infection, alloimmunization, febrile non-hemolytic effects, hemolysis, and transfusion-related acute lung injury (TRALI). Refractoriness to platelet transfusions resulting from HLA alloimmunization is a serious complication in up to 50% of patients, who permanently require hemotherapy with platelets. Another concern in cirrhotic patients results from the fact that transfused platelets are rapidly sequestered in the spleen leading to low increments. Consequently, transfusion therapy is only effective in the short term.

### 9.3. Prophylactic or “On-Demand” Platelet Transfusions

Facing with low platelet counts and the hemorrhagic risk, most physicians are inclined to reach “near-normal” platelet levels in patients with acute or chronic liver disease. Thus, prophylactic platelet transfusions are common practice “to warrant safety and optimal care”. However, as outlined above (Section 8 Laboratory Assessment), no evidence-based “trigger” for platelet transfusions exists. Consequently, guidelines from various societies provided only weak recommendations with regard to platelet count threshold levels at which “platelet transfusions should be considered” prior to scheduled invasive procedures. Very recent guidelines from the British Society of Gastroenterology, the Royal College of Radiologists, and the Royal College of Pathology and from the American Gastroenterology Association now define a uniform but still empirical “cut-off” with a platelet count of >50,000/μL as threshold needed prior to high-risk procedures/interventions [61,71]. Outside the interventional setting, no prophylactic platelet transfusions should be initiated to prevent patients from being subjected to unnecessary transfusions that provide no additional benefit [72].

By contrast, treatment with SDAPC or PPC remains a cornerstone of rescue therapy for active bleeding in patients with hepatic failure. As discussed below, additional plasma-derived or recombinant products such as fibrinogen concentrate or activated factor VIIa (rFVIIa) may be required to control severe hemorrhagic complications in the majority of patients.

### 9.4. Thrombopoietin (TPO) Receptor Agonists

The use of TPO receptor agonists/mimetics has provided substantial progress in the treatment of acquired thrombocytopenia in various conditions, including liver disease [25,73,74,75,76,77,78,79]. Among the four available agents, two of them, avatrombopag and lusutrombopag, received approval in 2018 by the Food and Drug Administration for treating thrombocytopenia in patients with chronic liver disease needing a scheduled invasive procedure [80,81]. Both second-generation TPO receptor agonists are orally administered drugs.

#### 9.4.1. Avatrombopag

In two seminal, identically designed, double-blind, randomized, placebo-controlled phase-3 trials, ADAPT-1 and ADAPT-2, avatrombopag was demonstrated to be superior to placebo in reducing the need for platelet transfusions or any rescue therapy for bleeding in 435 patients with thrombocytopenia and chronic liver disease undergoing a scheduled invasive procedure [82]. In both studies, patients were stratified into two groups based on lower (<40,000/μL) or higher (>40,000/μL to <50,000/μL) baseline platelet count levels. The efficacy of avatrombopag was documented in both cohorts with a significantly greater proportion in drug-treated than in corresponding placebo groups (Table 1). Upon administration of the TPO receptor agonist (40 or 60 mg daily for 5 days), platelet counts increased with a peak effect between days 10 and 13 and returned to baseline levels by day 35 [82]. The overall safety profile was similar for avatrombopag and placebo, except for hyponatremia reported as the most serious adverse effect.

#### 9.4.2. Lusutrombopag

Efficacy and safety of lusutrombopag were assessed in two randomized, double-blind, placebo-controlled trails, L-PLUS-1 and L-PLUS-2, enrolling 96 and 215 cirrhotic patients undergoing invasive interventions, respectively [83,84]. Key endpoints of the studies were avoidance of preprocedure platelet transfusions (L-PLUS-1 and -2), avoidance of rescue therapy for bleeding (L-PLUS-2), and number of days with platelet counts >50,000/μL (L-PLUS-2). Both trials showed that lusutrombopag (3 mg once daily for up to 7 days) was effective in achieving and maintaining the target platelet count in patients with thrombocytopenia (<50,000/μL) and chronic liver disease (Table 1). The median time to reach peak levels in platelet counts was 12 days, and the median duration of platelet levels over the threshold lasted 19 days [84]. No significant safety concerns were raised in both trials. The most common adverse effect of lusutrombopag was headache, while the most common serious adverse event was portal-vein thrombosis [84]. However, the number of thrombotic complications did not differ between drug- and placebo-treated patients (two in each group).

#### 9.4.3. Treatment Algorithm for the Management of Thrombocytopenia in Liver Disease

**Figure 2:**
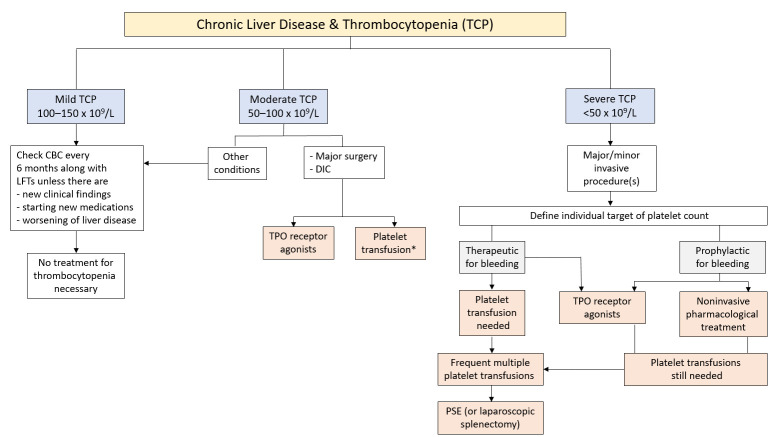
Proposed management of mild, moderate, and severe thrombocytopenia in patients with chronic liver disease. Displayed is the compilation of a treatment algorithm, originally designed by Gangireddy et al. [85] and subsequently adapted by Saab and Brown [25]. In this modification, administration of plasma products (e.g., fibrinogen concentrate, activated factor VII, prothrombin complex concentrate) as rescue therapy for active bleeding is omitted for clarity. To confirm no need for treatment, patients with mild thrombocytopenia should have periodic re-examination including complete blood screening. Of note, for moderate and severe thrombocytopenia, the use of TPO receptor agonists is now considered as first-choice treatment option, whereas platelet transfusions are restricted to major surgery and control of active hemorrhage. The star indicates that, upon platelet transfusion, a target platelet count >100,000/μL is aimed at. Abbreviations: CBC, complete blood cell count; DIC, disseminated intravascular coagulation; LFT, liver function testing; PSE, partial splenic embolization; TCP, thrombocytopenia; TPO, thrombopoietin.

In the light of new options given by the availability of safe and efficacious agents, Saab and Brown recently proposed a treatment algorithm [25] based on the original version by Gangireddy et al. [85]. Core elements of the updated modification (Figure 2) include (*i*) stratification of patients according to their degree of thrombocytopenia (with a “wait and re-examine” strategy for those with mild decrease in platelet count), (*ii*) administration of a TPO receptor agonist as first-line, and (*iii*) transfusion of platelet concentrates as second-line treatment or “back-up” option for high-risk patients, major surgery, or rescue therapy for patients with active hemorrhage.

It must be stressed, however, that the proposed treatment algorithm displayed in Figure 2 should be considered as a provisional recommendation, which requires careful evaluation and validation before becoming clinical practice. Specifically, caution is advised at this time regarding the use of TPO receptor agonists in patients with moderate thrombocytopenia when in critical conditions such as intracranial bleeding.

#### 9.4.4. Thrombotic Risk in Chronic Liver Disease Patients upon Treatment with TPO Receptor Agonists

Thrombotic events are a key safety concern with the use of either platelet transfusions or the administration of TPO receptor agonist to raise platelet counts in cirrhotic patients with severe thrombocytopenia. For example, a phase-3 trial using eltrombopag in this condition prior to invasive procedures was prematurely terminated because of an increased rate of thrombotic complications [27]. However, by contrast to subsequent trials evaluating second-generation TPO receptor agonists in chronic liver disease, eltrombopag was assessed by the ELEVATE (Eltrombopag Evaluated for its Ability to Overcome Thrombocytopenia and Enable Procedures) study group without abdominal imaging for splanchnic thrombosis at baseline. Thus, it is possible that some of the patients had a subclinical portal-vein thrombosis at study entry [27].

Several recent reviews and meta-analyses of studies (including more than 2200 patients in total) that compared the effect of three TPO receptor agonists (eltrombopag, avatrombopag, and lusutrombopag) and placebo in patients with chronic liver disease and thrombocytopenia reported on a trend toward increased risk of portal-vein thrombosis upon preprocedural treatment with the TPO receptor agonist (1.6% overall for the drugs vs. 0.6% for placebo) [25,86,87,88]. However, this difference was not statistically significant. Interestingly, a significant association between portal-vein thrombosis and TPO receptor agonist was shown for eltrombopag alone but not with avatrombopag or lusutrombopag treatments. In accord with these results, Michelson et al., reported that avatrombopag leads to an approximately two-fold increase in platelet counts but not in platelet activation among thrombocytopenic patients with liver disease [89].

Another analysis reviewed the number of arterial and venous thromboembolic events in more than 1700 patients treated with either eltrombopag or placebo in the preprocedural setting and identified a significantly higher rate of thromboembolic events in patients treated with eltrombopag (3.6%) than in those with placebo (1.1%) [25]. Overall, there are differences among oral TPO receptor agonists regarding their thrombotic potential, with eltrombopag carrying a significant thrombotic risk. However, this conclusion is questioned by others due to the fact that eltrombopag, as discussed above, was studied in the ELEVATE trial without appropriate pre-screening for portal-vein thrombosis [90].

#### 9.4.5. Medico-Economic Evaluation of Treatment with TPO Receptor Agonists vs. Platelet Transfusion

Several studies have assessed the clinical effectiveness in relation to the estimated cost-effectiveness of avatrombopag, lusutrombopag, and platelet transfusions. Some results of these analyses are equivocal or conflicting [91,92,93,94]. For example, Mladsi et al., reported that avatrombopag reduced the need for platelet transfusions and thus produced cost-savings compared with platelet transfusion (80% fewer prophylactic platelet transfusions, USD 4250 lower costs) or lusutrombopag (42% fewer platelet transfusions, USD 5820 lower costs) [91,92]. Others concluded from their cost-effectiveness analysis that avatrombopag and lusutrombopag are more expensive than no TPO receptor agonist over lifetime, as savings from avoiding platelet transfusions are exceeded by the drug cost and appear to be without long-term health benefits [93]. However, such a contention disregards that platelet transfusions are inappropriate as a sustained management option in patients with chronic liver disease.

## 10. Management of Hemostasis in Patients with Liver Disease

### 10.1. General Supportive Measures

Several general measures should be considered to stabilize or improve hemostasis in patients with liver disease, specifically in those with decompensated cirrhosis. Among others, these measures include: (*i*) control of the viral load (hepatitis B, C, or E virus), (*ii*) prevention and appropriate treatment of bacterial infection(s), (*iii*) therapy of renal dysfunction, and, importantly, (*iv*) avoidance of volume extension to reduce any side-effects on portal pressure and collateral vessels, in particular, varices. Indeed, understanding that volume extension increases portal pressure and promotes or exacerbates manifest bleeding in cirrhotic patients has had a significant impact on their management. Overall, this contention has promoted the concept of a “low-volume” approach to patients with chronic liver disease [31].

### 10.2. Additional Options for Supportive Hemotherapy to Bleeding Patients with Liver Disease

In accord with the “low-volume” concept but by contrast to common practice, a restrictive transfusion regimen is required in patients with liver disease to avoid prohemostatic hemotherapy and treatment-induced thrombotic complications or alloimmunization with resulting platelet refractoriness. Consequently, recent UK and US guidelines strongly recommend that blood products should be used sparingly in patients with acute or chronic liver disease [61,71].

Apart from the hemotherapy-induced increase of portal pressure, the risk of transfusion-associated circulatory overload, transfusion-related acute lung injury (TRALI), transmission of pathogens, alloimmunization, and/or transfusion reactions are major concerns. For management of active bleeding or high-risk invasive procedures the following transfusion thresholds are currently recommended to improve hemostasis in advanced liver disease: hematocrit >25%, platelet count >50,000/μL, and fibrinogen >120 mg/dL [61]. Of note, previously used thresholds for correction of the INR are no longer recommended since target reductions of INR are not supported by evidence.

#### 10.2.1. Fresh-Frozen Plasma (FFP)

Upon administration at common dosing (10 mL/kg), FFP has minimal effect on defective coagulation; only 10% of cirrhosis patients reach a reduction in INR [31]. The large volume of FFP required to reach an arbitrary INR target and to improve thrombin generation causes circulatory overload, thus limiting the indication and usefulness of FFP considerably.

#### 10.2.2. Prothrombin Complex Concentrates (PCCs) and/or Fibrinogen Concentrates

Replacement therapy with 4-factor (F) PCCs containing FII, FIX, FVII, and FX along with variable amounts of proteins C, S, and Z offers an attractive option to restore vitamin K-dependent coagulation factors with minimal volume.

In addition, whenever indicated (e.g., FI levels < 80 mg/dL), targeted substitution of fibrinogen using fibrinogen concentrates (e.g., Haemocomplettan^TM^, initial dosing 2 g or 30 mg/kg) should be considered and monitored by appropriate monitoring of coagulation and platelet function testing (e.g., by aggregometry). Two recent retrospective single-center studies confirm that hemotherapy with PCCs (and co-administration of fibrinogen concentrate) is safe and effective in patients with acute or chronic liver disease and liver transplantation [95,96]. Importantly, the rate of thromboembolic events in association with coagulation factor replacement therapy was less than 3% [95]. However, these treatment options must not be generalized.

By contrast, special caution and careful decision making are required. As discussed above (see Section 8 “Low Level” Hemostasis and Figure 1), patients with stable liver cirrhosis display a labile low-level equilibrium of pro- and anti-hemostatic components that is highly susceptible to pathogenic triggers such as inappropriate hemotherapy [8,16]. Specifically, decompensated liver disease can lead to increased consumption of coagulation factors, including progressive hypofibrinogenemia and progressive thrombocytopenia, often associated with hyperfibrinolysis [16,28]. Elevated levels of platelet, coagulation and fibrinolysis activation markers may be indicative of their defective clearance [29], mainly due to the reduced hepatocyte mass and a compromised monocyte-macrophage system, and/or reflect, in part, ongoing low-grade disseminated intravascular coagulation (DIC) [16,97]. Such a condition is prone to be boosted by inappropriate replacement therapy and eventually resulting in manifest DIC [11,97].

#### 10.2.3. Recombinant Activated Factor VII (rFVIIa)

rFVIIa is successfully used in a variety of conditions, including chronic liver disease, to control active hemorrhage [98,99,100]. Main settings related to end-stage liver disease are upper gastrointestinal bleeding (mainly from esophageal varices) and major surgery such as hepatectomy and liver transplantation. According to Consensus European Guidelines on the use of rFVIIa, this agent should not be administered to patients with Child-Pugh A cirrhosis; moreover, treatment with rFVIIa of acute hemorrhage in advanced liver disease (Child-Pugh B or C cirrhosis) is uncertain [101]. In addition, a number of thrombotic complications (stroke, myocardial ischemia, and portal-vein thrombosis) have been reported in patients with advanced liver disease following administration of rFVIIa, thus raising concerns on its safety in this setting (for which no license of rFVIIa exists) [99].

#### 10.2.4. Red Blood Cells (RBC)

Transfusion of packed RBC should also be managed restrictively due to adverse side effects on portal pressure, analogously to volume expansion upon administration of FFP, as outlined above. Current recommendations indicate a target hemoglobin of 7 to 8 g/dL [31]. Prior to scheduled invasive interventions, slightly higher hemoglobin levels (8.5 to 9.5 g/dL) should be achieved to improve hemostasis and thus reduce the risk of periprocedural complications [61].

### 10.3. Other Specific Agents

#### 10.3.1. Vitamin K

Replacement therapy can be considered in liver disease patients with an increased INR, which may in part reflect vitamin K deficiency (causing abrogated γ-carboxylation of coagulation factors II, VII, IX, and X and of proteins C and S). However, vitamin K deficiency is rather uncommon in this setting, unless there is coexisting cholestasis, antibiotic therapy, recent malnutrition, or long-term intensive care. If used, vitamin K should be administered parenterally due to impaired oral or intestinal adsorption in patients with liver failure. Caution is required, as intravenous application of vitamin K carries the risk of anaphylaxis.

#### 10.3.2. Desmopressin (1-Deamino-8-Arginine Vasopressin, DDAVP)

DDAVP is frequently used to improve hemostasis empirically. The agent can decrease prolonged bleeding time due to drug-induced acute release of von Willebrand factor from its endothelial storage organelles. The benefit of DDAVP administration in patients with liver disease is debated controversially. In fact, several randomized trials comparing DDAVP and placebo prior to invasive procedures (e.g., percutaneous liver biopsy; partial hepatectomy in patients with hepatocellular cancer) showed no difference in blood loss or transfusion requirements [31]. Overall, while side effects (e.g., headache, nausea, flush, diarrhea, or hyponatremia) from DDAVP are infrequent, and the agent is commonly used preprocedurally, its clinical efficacy has not been established in this setting.

#### 10.3.3. Antifibrinolytics

Antifibrinolytics may be useful when cirrhosis-associated hyperfibrinolysis is suspected or proven. Two agents are available, ε-aminocaproic acid (EACA) and tranexamic acid (TA), both of which abrogate binding of plasminogen or plasmin to fibrin, thus inhibiting fibrinolysis. There are only small series of patients and a number of case reports, demonstrating the efficacy and safety of EACA or TA in liver failure and advanced cirrhosis [102,103]. A systematic review and meta-analysis of more than 20 randomized controlled trials (with a total of 1400 patients) documented a reduction in RBC transfusions during liver transplantation when comparing TA to placebo, while the efficacy of EACA remained unproven in this setting (due an unpowered study) [104]. Importantly, this meta-analysis did not provide evidence for an increased risk of thromboembolic events associated with antifibrinolytic treatment in liver transplantation [104]. Overall, antifibrinolytic agents appear to be safe and well tolerated in cirrhotic patients [31].

## 11. Conclusions

Hemostatic dysfunction in acute and chronic liver disease and novel therapeutic options to control thrombocytopenia are a prime example for the significant progress that has been made in recent years. According to traditional paradigms, liver cirrhosis was considered as the epitome of an acquired coagulopathy that in combination with thrombocytopenia and/or thrombopathy causes hemorrhagic complications. Based on recent findings, this contention has been revised fundamentally in several aspects.

Firstly, patients with liver failure are also prone to thrombotic events, which may be even more common than bleeding complications, except for end-stage liver disease. Secondly, the commensurate decrease in pro- and antihemostatic components can lead to a *rebalanced low-level* hemostatic equilibrium. The rebalanced hemostatic system is, however, labile and can be destabilized by various triggers, which in turn may explain the occurrence of both bleeding and thrombotic complications. Thirdly, thrombocytopenia in liver cirrhosis results from multifaceted causes; apart from splenic pooling, decreased production of TPO plays a major role, as documented by the correlation between TPO levels and residual hepatic function. Fourthly, defective platelet function as a concomitant cause of bleeding events in hepatic failure has been overestimated in the past. However, drug-induced platelet inhibition remains an ongoing concern in this setting. Fifthly, high levels of plasma von Willebrand factor can restore platelet-vessel wall interaction and thus rebalance primary hemostasis that may be compromised otherwise in chronic hepatic disease. Sixthly, rebalanced hemostasis and, all the more, hypercoagulable features in liver disease have a major impact on the prevention and management of both bleeding and thrombosis.

This, however, is a challenging demand, which remains difficult to accompl in clinical practice. Most of routinely available hemostasis screening tests are inconclusive and without predictive validity in patients with liver disease, and comprehensive hemostasis profiles have to be restricted in this population to those undergoing high-risk invasive procedures such as major surgery.

Currently, the platelet threshold required for thrombocytopenic patients, who are scheduled for higher-risk interventions, is rather “defined” empirically but not based on study data of appropriate quality. However, recent advances are likely to solve this issue: the two newly licensed TPO receptor agonists, avatrombopag and lusutrombopag, were shown to be safe and efficacious at increasing platelet levels and avoiding platelet transfusions for invasive procedures in patients with thrombocytopenia and chronic liver disease. It can be expected that both agents will evolve to be the new standard of care for managing thrombocytopenia in patients with chronic liver disease.

## Figures and Tables

**Table 1:** Efficacy of second-generation TPO receptor agonists for the treatment of thrombocytopenia in patients with chronic liver disease scheduled for invasive procedures. Synopsis of results from four multicentric, randomized, double-blind, placebo-controlled phase-3 trials [82,83,84].

Agent	Trial	N of Patients (Drug/Placebo)	Dosing	Baseline Platelet Count	Outcome Percentage of Responders *	
Avatrombopag					Drug	Placebo
	ADAPT-1	*n* = 231 (149/82)	60 mg/d for 5 d	<40,000/L	65.6%	22.9%
			40 mg/d for 5 d	≧40,000/μL to <50,000/μL	88.1%	38.2%
	ADAPT-2	*n* = 204 (128/76)	60 mg/d for 5 d	<40,000/L	68.6%	34.9%
			40 mg/d for 5 d	≧40,000/μL to <50,000/μL	87.9%	33.3%
Lusutrombopag					Drug	Placebo
	L-PLUS-1	*n* = 96 (48/48)	3 mg/d for 7 d	<50,000/μL	79%	12.5%
	L-PLUS-2	*n* = 215 (108/107)	3 mg/d for 7 d	<50,000/μL	64.8%	29.0%

* Responders were defined as patients not requiring platelet transfusion or rescue therapy for bleeding through 7 days after the invasive procedure.
